# A Wetware Embodied AI? Towards an Autopoietic Organizational Approach Grounded in Synthetic Biology

**DOI:** 10.3389/fbioe.2021.724023

**Published:** 2021-09-23

**Authors:** Luisa Damiano, Pasquale Stano

**Affiliations:** ^1^ RG-ESA (Research Group on the Epistemology of the Sciences of the Artificial), Libera Università di Lingue e Comunicazione (IULM), Milan, Italy; ^2^ Department of Biological and Environmental Sciences and Technologies (DiSTeBA), University of Salento, Lecce, Italy

**Keywords:** artificial intelligence, artificial life, minimal cognition, embodied cognition, artificial cells, synthetic cells, synthetic biology

## Introduction

Embodied Artificial Intelligence (EAI) is a contemporary direction of AI characterized by developing its synthetic study of natural cognitive processes based on the assumption that the cognizer’s body plays a decisive role in cognition. In EAI the notion of “body” presents a wide range of interpretations, which, schematically, can be considered spanning between two extremes: the idea of an extra-neuronal material support for symbolic information processing, apt to ground symbols in sensori-motor associations; the concept of a multiple, integrated, environmentally embedded system, whose biological dynamics of self-organization are inseparable from (entangled with) processes of sense-making (e.g., Gallagher, 2011; Ziemke, 2016).

Often EAI is loosely identified with a robotic AI, that is, a form of AI targeting the construction and the experimental exploration of hardware models of natural cognitive processes. Indeed, electromechanical robots, unlike computers, are endowed with a body which situates them within the physical world — that is, not (only) within the abstract “informational world” — and allow them to interact with it based on sensors (e.g., sensors capable of detecting obstacles, light, sound, electromagnetic signals, etc.) and actuators. In the majority of the cases, EAI creates robots that are controlled by computers, in such a way that the robotic agent’s body grounds, in its sensori-motor interactions with the environment, the activity of the central processing unit, which functions as an information processing and a decision-making device. However, the EAI community is also engaged in building robots that are not guided by computers, and are capable of learning about their environments and accomplishing cognitive tasks only through their body (e.g., Brooks, 1991; Steels and Brooks, 1995).

Since its emergence in the early 1990s, EAI, through the variety of its expressions, has been realizing impressive advances, both at the fundamental and at the applicative research levels (e.g., Pfeifer and Bongard, 2006). Nonetheless, starting already in the late 1990s, debates have been raising concerns on the adequacy of EAI’s approach to the modeling of the biological body. Increasingly often these criticisms do not limit themselves to emphasize the mechanistic view of the body characterizing theories and implementations typical of EAI. They bring into attention EAI’s incapability of modeling the bodily organization, that is, the dynamic network of functional relations supporting the continuous self-production of the biological body through metabolism (Ziemke, 2016; Damiano and Stano 2018). These are radical criticisms, pointing out that currently EAI grounds its synthetic study of natural cognitive processes in a merely imitative modeling of the biological body: An artificial reconstruction that accounts only for superficial aspects of the bodily structure (e.g., movements and anatomical elements) and neglects its most specific dimension–its autonomous organization.

In this short article we intend to present the general programmatic lines of a research approach to EAI aiming at overcoming this gap. Such a program, in itself, is not a novelty. Research in EAI includes already “organizational programs,” such as organismic and enactive AI (e.g., Di Paolo, 2003; Froese and Ziemke, 2009), whose exploration of the role of the body in cognition intends to focus on the synthetic modeling of its organization. The specific originality of our organizational approach (Damiano and Stano, 2018) is that, while converging with the others in recognizing the chemical nature of the body’s organization, it diverges from them in pursuing its synthetic study of cognition based on building not hardware or software, but wetware — i.e., chemical — models of the biological body and its organization. In this sense, our approach proposes an unprecedented research program in EAI, which, in our view, represents one among the most challenging and, potentially, most rewarding research plans that could engage the efforts of the field in the next decades.

In the following pages we intend to present to the community the main programmatic lines of our approach, which are articulated around two cornerstones. The first is the methodological choice of developing our *wetware organizational EAI* by drawing on the technical and procedural advancements achieved, in the past two decades, by Synthetic Biology (SB). This research line in biology has been emerging since the 2000s as a new research direction in the “sciences of the artificial,” specialized into the chemical modeling of biological processes. The second choice, which is both epistemological and theoretical, is that of articulating the conceptual grounds of our SB approach to EAI in the theory of *autopoiesis,* i.e., the “biology of cognition” (Maturana 1970) developed, starting from the 1970s, by Humberto Maturana (1928–2021) and Francisco Varela (1946–2001), creative continuators of the tradition of experimental epistemology (McCulloch, 1965; Varela et al., 1991) to whose memory this commentary, and, more in general, our work in this field is dedicated.

## Prolegomena to a Wetware Autopoietic SB-AI

Grounding a research line of EAI in SB techniques and procedures means sketching a “SB-AI” research plan shaped not only by a theory of reference, but also by epistemological criteria and operative-technical specifications capable of guiding the experimental implementation. Here we intend to shortly delineate the preliminary concepts and approaches of our *wetware autopoietic SB-AI*, and the related programmatic framework. Our goal is not that of presenting a rigidly pre-defined research path. The intent of this short article is to propose to the community an explorative track, and to stimulate an open discussion on its potential developments.

### Embodied AI

EAI came about 30 years ago as a growing bush of research lines characterized by the common programmatic intent of overcoming the difficulties met by classical AI — i.e., computationalist AI, modeling cognitive processes in terms of programs for computers — by putting into focus the role of the body in cognition. Typically the related synthetic modeling of cognitive processes targets the construction of biologically inspired systems that, unlike computers, can learn about their environment and perform cognitively through their body. The programmatic idea is that of “complete” or “embodied” agents — in other words: robots, understood as artificial agents endowed with a body — whose cognitive abilities are generated by “emergent design,” that is, through interactions between different organizational levels. Generally, within EAI, these levels include the robotic agents as integrated systems, their subs-systems and their ecological niches — their environments and the systems constituting and populating them.

Since its first implementations, this approach has been proving to be very effective, as it has been allowing EAI to build increasingly autonomous and performant robotic agents. However, the ambition of building robots capable of cognitively interacting with their environment similarly to biological systems is still recognized out of EAI’s reach (e.g., Ziemke, 2016). According to pioneers and proponents of EAI engaged in the debate about the limits and the possibilities of the embodied approach to AI, the main obstacle is understanding and incorporating, in robotic agents, the “organizing principles of biological systems” (Brooks, 1997). As we discussed elsewhere (Damiano and Stano, 2018), overcoming this obstacle will lead to a qualitative shift in the synthetic modeling of biological systems: targeting not only superficial features of living systems, such as their movements and anatomical structures, but also the biological form of organization, viewed as the network of functional relations generating biological system’s self-production — their capability of producing their material identity by themselves, based on metabolism (Ziemke, 2001). Previous research programs, such as Organismically-inspired Robotics (Di Paolo, 2003) and Enactive AI (Froese and Ziemke, 2009), have proposed to address this challenge through organizational hardware and software models of biological systems, based on the autopoietic theoretical model of the (minimal) biological organization developed by Maturana and Varela (Varela et al., 1974; Maturana and Varela, 1980). Our research program converges with these approaches in adopting the autopoietic characterization of the biological organization, recognized accordingly as a chemical organization. However, our organizational approach to EAI diverges from these previous approaches in a key point: it pursues the synthetic recreation of the autopoietic organization through the construction of wetware — and not of hardware or software — implementations.

### Synthetic Biology

SB can be defined as the engineering-inspired branch of biology dedicated to design and build biological parts or systems not existing in nature, in order to use them for practical purposes (Endy, 2005; Andrianantoandro et al., 2006; de Lorenzo and Danchin, 2008). SB was born in the early 2000s and quickly grew as one of the most promising and challenging directions in science and technology. As it happens for AI and robotics, SB is often described as the science of the new century (Morange, 2009; Peccoud, 2016; Hockfield, 2019). Research in this field addresses issues as design, optimization and minimization, and spans from “rewiring” cell metabolism or regulatory networks to creating novel responsive molecular devices to control cell behavior, from grafting artificial sub-systems/modules into cells to extracting them for *in vitro* operations, and so on. Actually, SB is a bioengineering discipline characterized by a great ambition. The recent report on a cell controlled by a completely synthetic genome (designed and synthesized *ad hoc*) is perhaps one of the most paradigmatic example of SB progress. However, SB also deals with bottom-up approaches, that share with AI the understanding-by-building method, through which it engages in the (bio) chemical fabrication of living/living-like systems of minimal complexity. Typical targets are the modelling of living cells and living-like behavior by a total reconstruction, from scratch, of cell models (Luisi, 2002; Schwille et al., 2018). This is done by using biomolecules, or supposedly primitive compounds, or even artificial materials. Interesting hybrid combinations are also possible, as in the case of biological *organellae* inserted in artificial vesicles aiming at producing ATP (Altamura et al., 2021). Bottom-up SB, actually, can be considered the modern descendant of origins of life research, and counts as more proximal relatives the entire branch of chemical/biochemical reactions inside microcompartments, and the 1990s chemical autopoiesis (Bachmann et al., 1990; Walde et al., 1994). Typically, bottom-up SB research leaves aside the modeling of cognition, but it is actually endowed with the whole set of experimental and theoretical framework tools that could be deployed to successfully investigate that question.

### A SB Autopoietic Organizational Approach to EAI

Research programs like Organismically-inspired Robotics and Enactive AI certainly have high and recognized theoretical value, in terms of putting the biological organization at the center of their attention, and attempting to include it “on the experimental stage” of EAI. However, they have not yet produced concrete results. The programmatic idea, at the center of our research plan, is that of bringing the autopoietic organization on the experimental scene through a wetware autopoietic approach to EAI: A synthetic study of natural cognitive processes based on the construction and experimental exploration of wetware implementations of the autopoietic model of the biological organization.

The realization of our research program implies the introduction of the SB bottom-up synthetic cells techniques and procedures in EAI. This radical shift implies an opening towards new models, significantly different from current robotic models, and paves the way to radically new understanding of life and cognition, and — in the long run — radically new technologies. In particular, wetware-based approaches rely on chemical networks that are defined and propagate, at the same time, in the functional space and in the structural space, so that they actually self-generate autopoietic and embodied “agents.” The currently missing wetware approach to organizational AI calls for an intervention in this direction.

The current plan of implementation of our wetware autopoietic SB-AI research involves the integration of the following research dimensions.

1) The theoretical dimension. Translating the autopoietic theory of biological organization, and its filiations, in theoretical models that can be implemented in wetware models based on current SB techniques. Research questions: How can the autopoietic notion of biological organization be incoroporated in wetware systems like artificial cells and alike? How can the pivotal biological phenomenologies–i.e., life and cognition–be explored in synthetic chemical systems?
2) The experimental dimension. Developing of a bottom-up SB toolbox apt to build and experimentally explore these new AI wetware models. Research questions: What is the best material model for constructing (minimal) autopoietic systems? What is the path from simple models like autopoietic micelles (Luisi and Varela, 1989); or current non-autopoietic synthetic cells (Berhanu et al., 2019; Stano, 2019; Lavickova et al., 2020) to systems truly displaying autopoietic organizational relevant dynamics and AI features?
3) The epistemological dimension. Defining of a set of epistemological criteria to evaluate the relevance, for the scientific understanding of life and cognition, of organizational wetware models to be implemented. Research questions: How to evaluate the organizational relevance of synthetic models of natural (biological and cognitive) processes? What models could replace the popular, yet purely behavioral/imitative–not organizational (Damiano et al., 2011; Damiano and Stano, 2020)—Turing test?
4) The applicative dimension. Exploiting the resulting autopoietic cognitive synthetic cell technologies for applicative potentialities. Research questions: Can the SB-AI approach generate innovative engineering applications? Is there room for radically new technologies, in addition to the fairly obvious–yet challenging and surely rewarding–nanomedicine perspective?

## Concluding Remarks and Outlooks

Establishing a *wetware autopoietic organizational EAI* offers a possible way to address the limits of hardware-software EAI, and, more in general, of the sciences of artificial: Limiting the research to imitative models of biological and cognitive systems and processes. Here we promote a possible research approach to EAI, currently in development, which adopts the theory of autopoiesis as main starting conceptual framework of reference, and that exploits bottom-up SB synthetic cells technology as the main implementative approach of reference.

## DEDICATION

This article, and in general our work on this topic, is dedicated to the memory of Humberto Maturana (1928-2021) and Francisco J. Varela (1946-2001), who introduced the concept of autopoiesis in the 1970s.
